# Development of *Taenia pisiformis *in golden hamster (*Mesocricetus auratus*)

**DOI:** 10.1186/1756-3305-4-147

**Published:** 2011-07-25

**Authors:** Elizabeth Toral-Bastida, Adriana Garza-Rodriguez, Diego E Jimenez-Gonzalez, Ramon Garcia-Cortes, Guillermina Avila-Ramirez, Pablo Maravilla, Ana Flisser

**Affiliations:** 1 Hospital General "Dr. Manuel Gea Gonzalez", SSA. Calzada de Tlalpan 4800, Col Seccion XVI, Tlalpan, Mexico 14080 DF; 2 Departamento de Microbiologia y Parasitologia, Facultad de Medicina, Universidad Nacional Autonoma de Mexico, Mexico 04510 DF

## Abstract

The life cycle of *Taenia pisiformis *includes canines as definitive hosts and rabbits as intermediate hosts. Golden hamster (*Mesocricetus auratus*) is a rodent that has been successfully used as experimental model of *Taenia solium *taeniosis. In the present study we describe the course of *T. pisiformis *infection in experimentally infected golden hamsters. Ten females, treated with methyl-prednisolone acetate were infected with three *T. pisiformis *cysticerci each one excised from one rabbit. Proglottids released in faeces and adults recovered during necropsy showed that all animals were infected. Eggs obtained from the hamsters' tapeworms, were assessed for viability using trypan blue or propidium iodide stains. Afterwards, some rabbits were inoculated with eggs, necropsy was performed after seven weeks and viable cysticerci were obtained. Our results demonstrate that the experimental model of adult *Taenia pisiformis *in golden hamster can replace the use of canines in order to study this parasite and to provide eggs and adult tapeworms to be used in different types of experiments.

## Findings

Definitive hosts of *Taenia pisiformis *(Bloch, 1780) in nature are canines and rarely felines; lagomorphs and some rodents are intermediate hosts [1]. This tapeworm has been studied as an alternative model of taeniosis for those cestodes that are difficult to maintain under experimental conditions, such as *Taenia solium *and *Echinococcus*, since they can cause accidental human infections, or because they need specific requirements to develop [2]. Therefore, the development of an alternate experimental model of taeniosis that provides a reliable source of eggs as well as a trustworthy technique to assess the viability of these eggs would improve infection rates in vaccine trials and in the evaluation of novel anti-oncosphere drugs. It may also provide information on immune modulation in the gut due to worms that nowadays has become an important research area [3,4].

Previous studies indicate that only rodents from Cricetidae, Heteromyidae and Chinchillidae families are susceptible to adult cestode experimental infections [5,6]. In addition, administration of steroids to rodents has resulted in a higher recovery rate of the worms as well as exhibition of a greater sexual development and survival for longer periods [7-10]. The golden hamster (*Mesocricetus auratus*) has been used to develop mature or gravid *Diphyllobothrium sp*. [11], *Echinococcus multilocularis *[5], *Taenia solium *[7-10] and *T. crassiceps *[12,13] tapeworms. Nevertheless, the production of viable *T. pisiformis *eggs in an unnatural definitive host has not been previously documented. Thus, the objective of the present study was to describe the course of *T. pisiformis *infection in golden hamsters.

Prior to experiments, the Internal Committee for the Use and Care of Laboratory Animals of the General Hospital "Dr. Manuel Gea Gonzalez", which reviews the ethical and research aspects of protocols that include animals, approved this study. A minimum number of animals required to obtain valid results were used, according to the statements of the American Association for Laboratory Animal Science [14]. Ten New Zealand female white rabbits, two to three months old and 10 female golden hamsters three months old, were hosted individually in metal battery-style caging with hardwood chips as bedding and standard polycarbonate cages for rabbits and hamsters. Cages were set in a climate controlled windowless indoor room and animals were fed with commercial pellets and water *ad libitum*. Two weeks before infection, hamsters were treated with a single dose of 30 mg/kg body weight of praziquantel (Cesol, Merck).

Seven *Taenia pisiformis *adult worms were recovered during necropsy from a stray hybrid dog used for training at the Department of Pathology, Faculty of Veterinary Medicine, National University of Mexico. Parasite species was confirmed by the morphology of rostelum hooks and by identification of gravid proglottids stained with Mayer's Carmine. Tapeworms were washed exhaustively with PBS and preserved for one week at 4°C in PBS supplemented with penicillin G (1,000 IU/ml) and streptomycin sulphate (10 mg/ml). Eggs were obtained as described by Coman and Rickard [15]; briefly, terminal segments from each gravid worm were cut into small fragments and filtered through a sieve (150 μm pores), washed with PBS, centrifuged to 2500 rpm for 10 min, and counted using a Neubauer chamber. Eggs were pooled from all tapeworms recovered and aliquots were used to assess oncosphere viability and to infect rabbits. For hatching, 500 μl of egg solution (with approximately 5000 mature eggs) were poured per well in a 24 well culture cell plate, 1 ml of sodium hypochlorite stock solution (0.5% in PBS) were added and gently mixed with a Pasteur pipette; disruption of embryophoric blocks was followed under an inverted microscope. When most oncospheres were released from their embryophores, they were transferred to a conical plastic tube, PBS was added and the suspension centrifuged at 1,000 *g *for 5 min, according to Wang et al. [16]. The pellet containing oncospheres was washed, re-suspended in 1 ml PBS, and placed in two wells of one plate. For the *in vitro *oncosphere viability assay 1:10 solution of 0.4% trypan blue solution (TB; Sigma) or 0.001% propidium iodide solution (PI; Sigma) was added to the wells and was gently mixed with a Pasteur pipette. Viability was determined 5 min later by counting stained and unstained oncospheres under light microscopy for TB, and under UV light for IP. After oncosphere viability assessment, five rabbits were infected orally through a stomach tube with 1500 *T. pisiformis *mature eggs each according to Rickard and Outteridge [17] and humanely euthanized nine weeks later. Cysticerci were recovered from their peritoneal cavities, washed in PBS, measured and maintained on ice. Under a stereoscopic microscope, the parasites' vesicular membranes were cut to eliminate the vesicular fluid avoiding damage to the scolex, in this way facilitating their swallowing during hamster infection. Rodents were given three cysticerci orally; that had all the characteristics of a live parasite (round, clear with vesicular fluid and an invaginated scolex). Treatment with methyl-prednisolone acetate (MPA, Depomedrol, Up-John, Mexico) began on the day of infection with 2 mg per hamster every two weeks post-inoculation (WPI) [6]. After three WPI, recovered faeces were hydrated for one day, homogenized and sieved to identify proglottids, which were washed exhaustively with water and then with PBS and analysed under a light microscope. All proglottids released by the hamsters were preserved at 4°C until stained and mounted in order to identify their maturation. At 16 WPI, hamsters were euthanized, intestines were opened longitudinally and tapeworms were recovered, washed with PBS and kept at 4°C.

Egg viability was assessed by TB or IP dyes as described above. Five rabbits were orally inoculated with 1500 eggs, necropsies were performed at seven WPI and recovered cysticerci were counted and stained with Mayer's Carmine. Data are expressed as percentages (%), mean and standard deviation. Mantel-Haenszel and Fisher exact two-way, tests were performed with Epi-Info6 v6.04 software.

Length and development of *T. pisiformis *adults and cysticerci are summarized in Table 1. Two mature and five gravid tapeworms, measuring 41 to 79 cm were recovered from the dog. All hamsters became infected (according to the presence of proglottids released in faeces or tapeworms at necropsy); at the end of study only four rodents were parasitized. To avoid suffering of hamsters caused by MPA and to study the development of the tapeworms, rodents were euthanized according to the recommendations of the American Veterinary Medical Association [18] and worms recovered were placed in distilled water and stored overnight at 4°C for relaxation and measuring.

**Table 1:** Data of recovery, length and development of *T. pisiformis *adults and cysticerci during infections in dog, hamsters and rabbits

	Parasitized host	Number of animals used	Number of infected animals (%)	Total parasites recovered/number of animals parasitized at necropsy	Features of recovered parasites	
					
					Development	Length (cm)
Taeniosis	Dog	1	1 (100)	7/1	Mature Gravid	13,15 41,54,57,59,79
	Hamsters	10	10 (100)	6/4	Immature Mature Gravid Gravid	3,6 10,12 21 25
Cysticercosis	Rabbits (eggs from the dog tapeworms)	5	4 (80)	47/4	5 non-developed 2 viable/5 non-developed 10 viable/3 non-developed 19 viable/3 non-developed	0.3 0.9-1.1/0.3-0.5
	Rabbits (eggs from hamster tapeworms)	5	2 (40)	8/2	2 non-developed 6 viable	0.3 0.9-1.1

A total of 47 cysticerci (31 viable and 16 non-developed, seen as white fusiform spots in the liver) were recovered from the rabbits, thus 0.63% infection efficiency (7500 eggs were used to infect five rabbits) was obtained. In contrast when eggs recovered from two gravid tapeworms obtained from the hamsters were used to infect five rabbits, only two became parasitized with eight cysticerci recovered (six viable from one rabbit and two non-developed from the other one), yielding 0.11% infection efficiency. No statistically differences were found between rabbit infections using eggs from the dog or the hamsters tapeworms (*p *= 1.00, Fisher exact test).

Proglottids with different degree of development were intermittently released in faeces from three WPI, while gravid proglottids segments were seen from nine WPI. A small number of mature eggs per proglottid were identified in the two gravid tapeworms recovered from hamsters that were not counted to avoid loss during handling. Viability (defined as non-stained organisms) was 84% by TB and 94% by IP in 795 and 630 oncospheres obtained from the dog tapeworm, while 70% by TB and 98% by IP in 60 and 69 oncospheres from hamsters' worms. Non-viable oncospheres were clearly different since they were blue in TB and red in IP. Statistical differences were found between viability of eggs from the dog and the hamsters' tapeworms only by TB (*p *= 0.005, Mantel-Haenszel test).

The use of animal models for the study of taeniosis due to *Taenia *species has been critical for understanding the host-parasite relationship during infection; thus, earlier studies focused to assess the immune response and biological features of *T. pisiformis*, were performed using dogs and rabbits as natural definitive and intermediate hosts respectively [19-21]. Our data show that *T. pisiformis *is capable of establishing and developing into gravid adult worms with infective eggs in golden hamsters treated with MPA. Similarly, as with taeniosis due to *T. solium*/*Chinchilla laniger*, the gravid tapeworms recovered from an unnatural definitive host are smaller and with less mature eggs than those recovered from natural hosts [6,22]; in contrast, during experimental taeniosis by *T. crassiceps *in hamsters treated with prednisolone, the egg number was apparently higher than those found in dogs [23].

Trypan blue and propidium iodide were both useful dyes to assess oncosphere viability in experimental infections with *T. solium *eggs obtained from chinchillas [22]; however, in the present study, TB had a better match with infectivity and was technically easier to perform, since a UV light microscope is not necessary to observe the stain. Our results demonstrate that the experimental model of *Taenia pisiformis *in golden hamster is an alternative for providing eggs and adult tapeworms to be used in different types of experiments,.

## Abbreviations

MPA: methyl-prednisolone acetate; WPI: weeks post-inoculation; TB: trypan blue; PI: propidium iodide.

## Competing interests

The authors declare that they have no competing interests.

## Authors' contributions

AF and PM formulated the idea and wrote the manuscript, ETB, AGR, DEJG and RGC performed the experimental processes. GAR provided critical comments to the protocol and the discussion. All authors approved the final version of the manuscript.
